# Association Between Depressive Symptoms and Severe Vision Difficulty Among United States Adults

**DOI:** 10.7759/cureus.106098

**Published:** 2026-03-30

**Authors:** Jingwen Wang, Jia Qu, Ying Li

**Affiliations:** 1 Department of Ophthalmology, Peking Union Medical College Hospital, Chinese Academy of Medical Sciences and Peking Union Medical College, Beijing, CHN; 2 Research Unit of Myopia Basic Research and Clinical Prevention and Control, Chinese Academy of Medical Sciences (2019RU025), Wenzhou, CHN; 3 School of Optometry and Ophthalmology and Eye Hospital, Wenzhou Medical University, Beijing, CHN

**Keywords:** cross-sectional study, depressive symptoms, nhanes, phq-9, severe vision difficulty, vision impairment

## Abstract

Background: Depression and vision impairment are major public health concerns, yet the association between depressive symptom severity and severe vision difficulty remains unclear.

Methods: We conducted a cross-sectional study using data from the National Health and Nutrition Examination Survey 2013-2018. A total of 9,227 adults were included. Depressive symptoms were assessed using the Patient Health Questionnaire-9. Severe vision difficulty was defined as self-reported serious difficulty seeing or being blind. Survey-weighted logistic regression, restricted cubic spline modeling, and subgroup analyses were performed to evaluate the associations.

Results: Among 9,227 participants, 632 (6.85%) reported severe vision difficulty. In the fully adjusted model, compared with participants with no depressive symptoms, the odds ratios for severe vision difficulty were 1.74 (95% CI: 1.28-2.37) for mild depressive symptoms, 2.28 (95% CI: 1.56-3.34) for moderate depressive symptoms, 4.77 (95% CI: 2.16-10.51) for moderately severe depressive symptoms, and 5.20 (95% CI: 2.87-9.44) for severe depressive symptoms (P for trend < 0.001). When analyzed continuously, each five-point increase in PHQ-9 score was associated with higher odds of severe vision difficulty (OR: 1.60, 95% CI: 1.43-1.79, P < 0.001). Restricted cubic spline modeling showed a significant overall association (P for overall association < 0.001), whereas the test for nonlinearity was not significant (P for nonlinearity = 0.560). Exploratory subgroup analyses showed no significant interaction across major demographic and clinical strata (all P for interaction > 0.05).

Conclusions: Depressive symptom severity was independently and linearly associated with severe vision difficulty among adults in the United States. These findings suggest that greater depressive symptom burden may be linked to poorer visual health in the general population.

## Introduction

Visual impairment and depressive symptoms are major public health challenges that substantially contribute to disability and reduced quality of life worldwide [1,2]. These conditions are common, burdensome, and often coexist, potentially compounding limitations in functional status, daily activities, and overall well-being. Accordingly, a clearer understanding of the association between depressive symptom severity and severe vision difficulty may have important clinical and public health relevance.

Previous epidemiological studies have reported a close association between visual impairment and adverse mental health outcomes [3,4]. Individuals with vision loss often experience reduced independence, social isolation, and diminished quality of life, factors that may be associated with greater depressive symptom burden. Longitudinal and population-based studies have shown that people with visual impairment are more likely to report depressive symptoms, psychological distress, and poorer mental health status [5-7].

However, most existing studies have focused on visual impairment as a predictor of depressive outcomes, emphasizing how vision loss may be associated with greater depressive symptom burden. By contrast, fewer population-based studies have examined whether increasing depressive symptom severity is associated with severe vision difficulty. In addition, many prior studies have treated depression-related measures as a binary condition, which may obscure potential gradients across levels of symptom severity. Consequently, the association between depressive symptom severity and severe vision difficulty has not been fully characterized in large population-based samples.

To address these gaps, we used data from the National Health and Nutrition Examination Survey (NHANES) 2013-2018, a nationally representative survey of adults in the United States. The primary objective of this study was to examine the association between depressive symptom severity, assessed using the Patient Health Questionnaire-9 (PHQ-9), and severe vision difficulty among U.S. adults. Secondary analyses evaluated this relationship using both categorical and continuous PHQ-9 measures and explored its functional form with restricted cubic spline modeling. We hypothesized that higher depressive symptom severity would be independently associated with greater odds of severe vision difficulty.

## Materials and methods

Study population and data source

This study used data from the NHANES 2013-2018, a nationally representative cross-sectional survey of the non-institutionalized United States population conducted by the Centers for Disease Control and Prevention. A total of 29,400 participants were identified from the 2013-2018 survey cycles. Participants with missing data on severe vision difficulty were excluded (n = 1,168), leaving 28,232 participants. Individuals with missing covariate data were subsequently excluded from the base analytic sample (n = 19,005), resulting in 9,227 participants for analysis. The NHANES protocol was approved by the National Center for Health Statistics Ethics Review Board, and all participants provided written informed consent at the time of the original survey. Because the present study was a secondary analysis of de-identified, publicly available data, no additional informed consent was required.

Outcome assessment: severe vision difficulty

Severe vision difficulty was assessed using the NHANES disability questionnaire administered during the in-home household interview by trained interviewers. Participants were asked whether they were blind or had serious difficulty seeing even when wearing glasses. Those responding “yes” were classified as having severe vision difficulty, whereas those responding “no” were classified as not having severe vision difficulty. Responses indicating refusal, uncertainty, or missing data were excluded. We retained this binary endpoint because it is the directly available vision-related functional measure in NHANES 2013-2018 and because its wording is closely aligned with the American Community Survey standard vision disability item, facilitating comparability with population-based disability surveillance. This dichotomous measure has been widely used in NHANES-based ophthalmic epidemiological studies [8-10].

Exposure assessment: depressive symptoms

Depressive symptoms were assessed using the PHQ-9, a self-reported instrument that evaluates the frequency of depressive symptoms during the previous two weeks [11-14]. For categorical analyses, PHQ-9 scores were classified as no depressive symptoms (0-4), mild depressive symptoms (5-9), moderate depressive symptoms (10-14), moderately severe depressive symptoms (15-19), and severe depressive symptoms (20-27). The no depressive symptoms group was used as the reference category. For continuous analyses, the PHQ-9 score was additionally modeled per five-point increase.

Covariates

Potential confounders were selected on the basis of clinical relevance, previous literature, and consistent availability across the NHANES 2013-2018 cycles. These covariates included age, sex, race and ethnicity, education level, poverty-income ratio, body mass index (BMI), diabetes, hypertension, smoking status, physical activity, and laboratory measures. Race and ethnicity were self-reported in NHANES and were included as sociodemographic covariates. Diabetes was defined as a self-reported physician diagnosis, current use of glucose-lowering medication, a glycated hemoglobin level of 6.5% or greater, or a fasting plasma glucose level of 126 mg/dL or greater. Hypertension was defined as a self-reported physician diagnosis, current use of antihypertensive medication, or measured blood pressure with systolic blood pressure ≥130 mmHg and/or diastolic blood pressure ≥80 mmHg. Measured blood pressure was obtained in the mobile examination center (MEC) after a five-minute seated rest, and up to three seated blood pressure readings were averaged. Smoking status was categorized as never, former, or current.

Physical activity was derived from the NHANES physical activity questionnaire, which is based on the Global Physical Activity Questionnaire framework and includes work/household, transportation, and leisure-time domains. Total physical activity was calculated as crude weekly minutes, rather than MET-minutes, by summing the products of reported days per week and minutes per day across vigorous work activity, moderate work activity, walking or bicycling for transportation, vigorous leisure-time activity, and moderate leisure-time activity. Participants were categorized as having low (<150 min/week), moderate (150-299 min/week), or high (≥300 min/week) physical activity [15].

Weighting and survey design

NHANES uses a complex, multistage probability sampling design. All analyses incorporated survey weights, strata, and primary sampling units in accordance with NHANES analytic guidance. Because the analyses included examination- and laboratory-derived variables, MEC examination weights were used. For pooled analyses across the 2013-2014, 2015-2016, and 2017-2018 cycles, six-year MEC weights were constructed by dividing the two-year MEC examination weights by three. Variance estimation incorporated SDMVSTRA and SDMVPSU using Taylor series linearization.

Statistical analysis

Baseline characteristics were compared between participants with and without severe vision difficulty using survey-weighted t-tests for continuous variables and Rao-Scott-adjusted chi-square tests for categorical variables. Continuous variables are presented as means with standard deviations, and categorical variables are presented as weighted percentages. Survey-weighted logistic regression models were used to estimate odds ratios (ORs) and 95% confidence intervals (CIs). Three models were constructed. Model 1 was unadjusted. Model 2 was adjusted for age, sex, and race and ethnicity. Model 3 was further adjusted for education level, poverty-income ratio, smoking status, BMI, hypertension, diabetes, and physical activity.

Restricted cubic spline models were fitted using the raw PHQ-9 score to examine the shape of the association between depressive symptom severity and severe vision difficulty. The spline model used internal knots at 5, 10, and 15, with boundary knots at 0 and 27. Subgroup analyses and interaction tests were performed across major demographic and clinical strata. All tests were two-sided, and P-values less than 0.05 were considered statistically significant. All statistical analyses were performed using R version 4.4.2 (R Foundation for Statistical Computing, Vienna, Austria).

## Results

Participant characteristics

**Figure 1:**
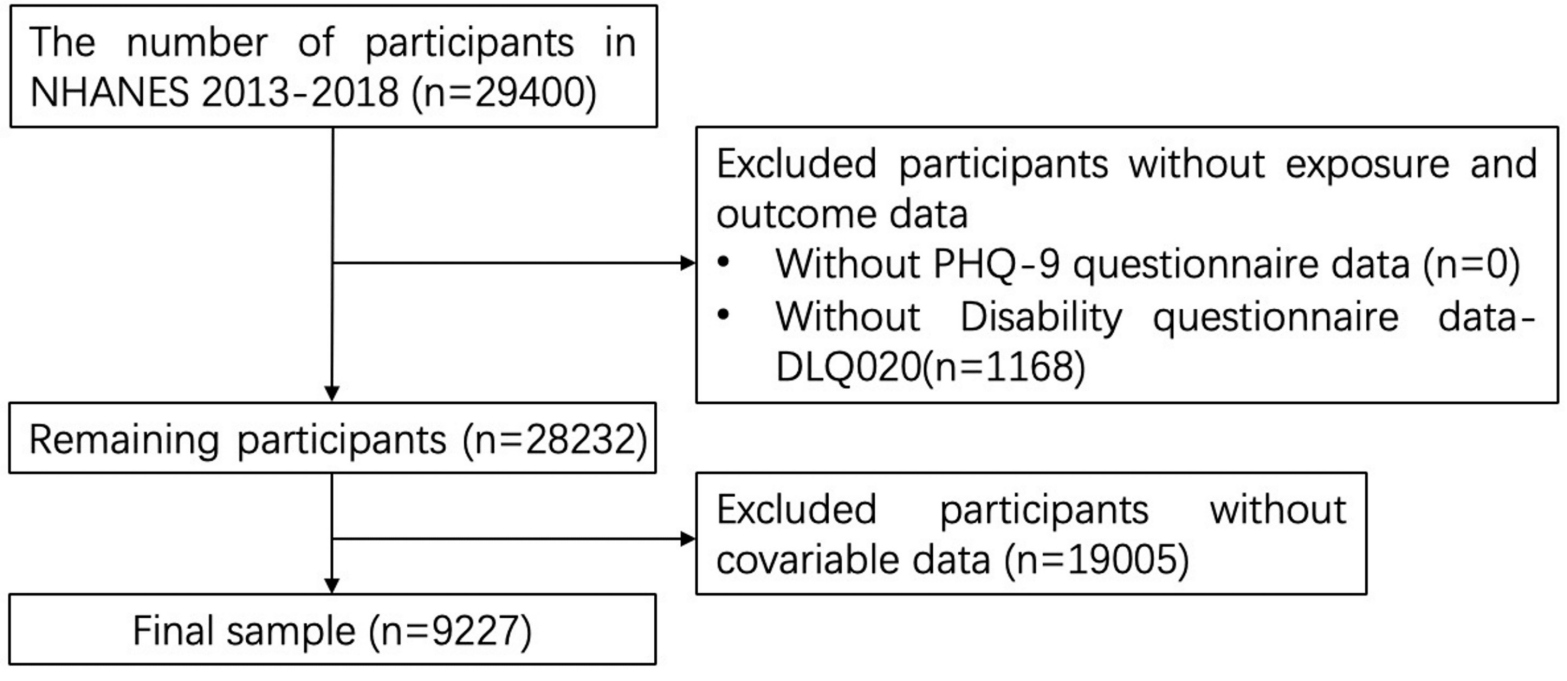
Flowchart of the study population selection process NHANES 2013-2018 participant selection process. NHANES: National Health and Nutrition Examination Survey; PHQ-9: Patient Health Questionnaire-9.

A total of 9,227 participants were included in the final analysis, as shown in Figure 1.

Table 1 summarizes the baseline characteristics of the study population based on severe vision difficulty status. The overall mean age of the participants was 46.06 ± 19.17 years. Individuals with severe vision difficulty were significantly older than those without severe vision difficulty (55.46 ± 17.91 vs. 45.49 ± 19.09 years, P < 0.001). They also had a higher prevalence of depression (21% vs. 6.7%, P < 0.001), diabetes (47% vs. 23%, P < 0.001), and hypertension (73% vs. 50%, P < 0.001). In addition, participants with severe vision difficulty were more likely to have lower educational attainment (33% vs. 19% with less than a high school education, P < 0.001), lower poverty-income ratio levels (26% vs. 15% with a poverty-income ratio < 1, P < 0.001), and lower physical activity levels (54% vs. 42% with low activity, P = 0.004).

**Table 1:** Baseline characteristics of the study population Continuous variables are presented as weighted mean ± standard deviation, and categorical variables are presented as unweighted counts with weighted percentages. P-values were calculated using survey-weighted t tests for continuous variables and Rao-Scott-adjusted chi-square tests for categorical variables. NHANES: National Health and Nutrition Examination Survey; PIR: poverty-income ratio; BMI: body mass index; HbA1c: hemoglobin A1c.

Variables	Overall, n = 9227	No severe vision difficulty, n = 8595	Severe vision difficulty, n = 632	P-value
Age (years)	46.06 ± 19.17	45.49 ± 19.09	55.46 ± 17.91	<0.001
Gender (%)				
Female	4,705 (51)	4,369 (51)	336 (56)	0.129
Male	4,522 (49)	4,226 (49)	296 (44)	
Race (%)				
Non-Hispanic White	3,389 (65)	3,169 (65)	220 (61)	0.001
Mexican American	1,459 (9.3)	1,325 (9.1)	134 (12)	
Non-Hispanic Black	1,962 (11)	1,833 (11)	129 (12)	
Other Hispanic	925 (5.8)	830 (5.7)	95 (8.6)	
Other Race	1,492 (9.3)	1,438 (9.5)	54 (5.8)	
BMI	29.34 ± 7.48	29.27 ± 7.45	30.54 ± 7.90	0.002
Education (%)				
Below High School	1,746 (20)	1,498 (19)	248 (33)	<0.001
High School	1,759 (33)	1,613 (33)	146 (36)	
Above High School	2,412 (46)	2,281 (48)	131 (31)	
PIR Level (%)				
<1	2,203 (16)	1,970 (15)	233 (26)	<0.001
≥1	7,024 (84)	6,625 (85)	399 (74)	
HbA1c (%)	5.81 ± 1.17	5.77 ± 1.12	6.46 ± 1.70	<0.001
Diabetes (%)				
No	6,509 (76)	6,214 (77)	295 (53)	<0.001
Yes	2,718 (24)	2,381 (23)	337 (47)	
Hypertension (%)				
No	3,695 (48)	3,545 (50)	150 (27)	<0.001
Yes	4,624 (52)	4,168 (50)	456 (73)	
Smoking (%)				
Never	4,609 (55)	4,335 (56)	274 (42)	<0.001
Former	2,028 (27)	1,844 (26)	184 (34)	
Current	1,478 (18)	1,336 (17)	142 (25)	
Activity (%)				
Low	4,388 (42)	4,001 (42)	387 (54)	0.004
Moderate	1,109 (13)	1,048 (13)	61 (11)	
High	3,730 (45)	3,546 (46)	184 (35)	
Depression (%)				
No	8,504 (92)	8,012 (93)	492 (79)	<0.001
Yes	723 (7.5)	583 (6.7)	140 (21)	

Association between depressive symptom severity and severe vision difficulty

The association between depressive symptom severity and severe vision difficulty was evaluated using survey-weighted logistic regression models (Table 2). In the unadjusted model, a graded association was observed across increasing PHQ-9 categories. Compared with participants with no depressive symptoms, the odds ratios for severe vision difficulty were 2.30 (95% CI, 1.76-3.01) for mild depressive symptoms, 3.16 (95% CI, 2.21-4.52) for moderate depressive symptoms, 7.26 (95% CI, 3.88-13.57) for moderately severe depressive symptoms, and 7.03 (95% CI, 4.16-11.90) for severe depressive symptoms (P for trend < 0.001). After adjustment for age, sex, and race/ethnicity, the association remained statistically significant. In the fully adjusted model, the graded relationship persisted. Compared with participants with no depressive symptoms, the odds ratios for severe vision difficulty were 1.74 (95% CI, 1.28-2.37) for mild depressive symptoms, 2.28 (95% CI, 1.56-3.34) for moderate depressive symptoms, 4.77 (95% CI, 2.16-10.51) for moderately severe depressive symptoms, and 5.20 (95% CI, 2.87-9.44) for severe depressive symptoms (P for trend < 0.001).

**Table 2:** Association between depressive symptom severity and severe vision difficulty Model 1 was unadjusted. Model 2 was adjusted for age, sex, and race and ethnicity. Model 3 was additionally adjusted for education level, poverty-income ratio, smoking status, body mass index, hypertension, diabetes, and physical activity. All models accounted for the complex survey design of NHANES. NHANES: National Health and Nutrition Examination Survey; OR: odds ratio; CI: confidence interval.

Model	No depressive symptoms (0–4)	Mild depressive symptoms (5–9)	Moderate depressive symptoms (10–14)	Moderately severe depressive symptoms (15–19)	Severe depressive symptoms (20–27)	P for trend
Model 1	1	2.30 (1.76–3.01)	3.16 (2.21–4.52)	7.26 (3.88–13.57)	7.03 (4.16–11.90)	<0.001
Model 2	1	2.01 (1.55–2.61)	2.85 (1.96–4.14)	6.32 (3.35–11.93)	6.80 (4.03–11.47)	<0.001
Model 3	1	1.74 (1.28–2.37)	2.28 (1.56–3.34)	4.77 (2.16–10.51)	5.20 (2.87–9.44)	<0.001

When the PHQ-9 score was analyzed as a continuous variable, each five-point increase was associated with higher odds of severe vision difficulty in the fully adjusted model (OR, 1.60; 95% CI, 1.43-1.79; P < 0.001), as shown in Table 3.

**Table 3:** Association between Patient Health Questionnaire-9 score and severe vision difficulty per five-point increase Associations are presented per five-point increase in the Patient Health Questionnaire-9 score. Model 1 was unadjusted. Model 2 was adjusted for age, sex, and race and ethnicity. Model 3 was additionally adjusted for education level, poverty-income ratio, smoking status, body mass index, hypertension, diabetes, and physical activity. All models accounted for the complex survey design of NHANES. OR: odds ratio; CI: confidence interval; NHANES: National Health and Nutrition Examination Survey.

Model	OR (95% CI)	P for trend
Model 1	1.78 (1.63–1.94)	<0.001
Model 2	1.72 (1.58–1.88)	<0.001
Model 3	1.60 (1.43–1.79)	<0.001

Restricted cubic spline analysis

**Figure 2:**
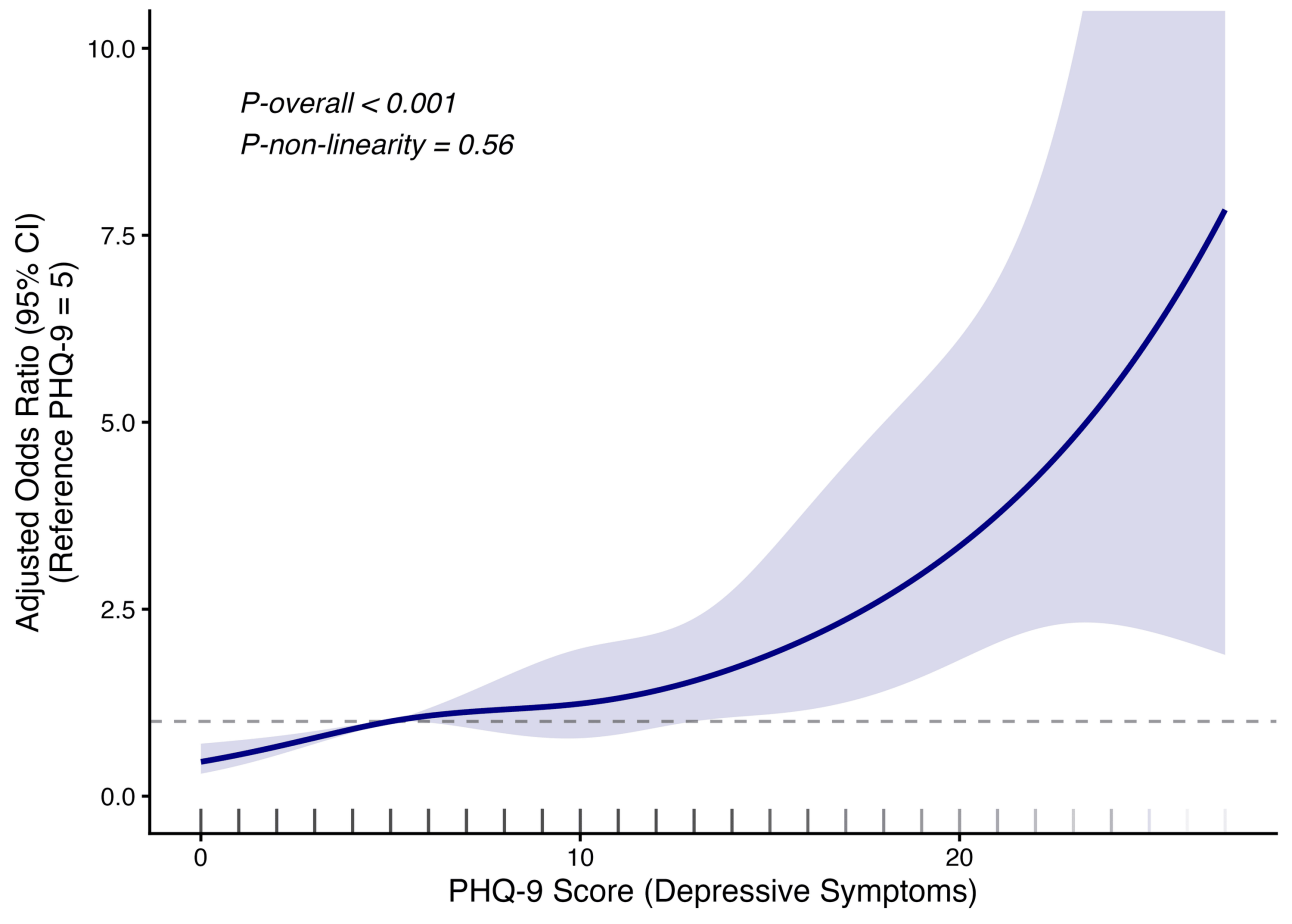
Association between Patient Health Questionnaire-9 score and severe vision difficulty based on restricted cubic spline modeling Restricted cubic spline model showing the association between Patient Health Questionnaire-9 score and severe vision difficulty. The solid line represents adjusted odds ratios, and the shaded area represents 95% confidence intervals. The model was adjusted for age, sex, race and ethnicity, education level, poverty-income ratio, smoking status, body mass index, hypertension, diabetes, and physical activity. PHQ-9: Patient Health Questionnaire-9; CI: confidence interval.

Restricted cubic spline modeling was performed to further examine the association between PHQ-9 score and severe vision difficulty (Figure 2). A statistically significant overall association was observed (P overall < 0.001). However, the test for non-linearity was not significant (P for non-linearity = 0.560), indicating limited evidence of meaningful curvature and suggesting that the association was largely close to linear across the observed PHQ-9 range.

Subgroup analyses

Exploratory subgroup analyses were performed to assess whether the association between PHQ-9 score and severe vision difficulty differed across major demographic and clinical strata (Table 4). Positive associations were observed across most subgroups. However, no statistically significant interaction was detected for age, BMI, diabetes, education level, sex, hypertension, poverty-income ratio, physical activity, race/ethnicity, or smoking status (all P for interaction > 0.05). These findings suggest that the direction of association was generally similar across strata, although the subgroup analyses should be interpreted as exploratory.

**Table 4:** Subgroup analyses of the association between Patient Health Questionnaire-9 score Models were adjusted as appropriate within each subgroup. P for interaction represents the significance of interaction across subgroups. Age is expressed in years, and BMI is expressed in kg/m². OR: odds ratio; CI: confidence interval; PHQ-9: Patient Health Questionnaire-9; BMI: body mass index; PIR: poverty-income ratio.

Subgroup	Sample size	OR (95% CI)	P-value	P for interaction
Age (years)				
<60	6235	1.60 (1.40–1.84)	<0.001	0.583
≥60	2992	1.59 (1.30–1.95)	<0.001	
BMI (kg/m²)				
<30	5676	1.75 (1.48–2.06)	<0.001	0.177
≥30	3551	1.47 (1.29–1.68)	<0.001	
Diabetes				
No	6509	1.60 (1.39–1.85)	<0.001	0.935
Yes	2718	1.57 (1.37–1.80)	<0.001	
Education Level				
Below High School	1746	1.72 (1.48–2.00)	<0.001	0.11
Above High School	2412	1.76 (1.44–2.16)	<0.001	
High School	1759	1.35 (1.08–1.69)	0.01	
Gender				
Female	4705	1.65 (1.44–1.89)	<0.001	0.867
Male	4522	1.55 (1.21–1.98)	0.001	
Hypertension				
No	3695	1.68 (1.28–2.19)	<0.001	0.569
Yes	4624	1.55 (1.38–1.74)	<0.001	
PIR Level				
<1	2203	1.39 (1.19–1.62)	<0.001	0.067
≥1	7024	1.70 (1.48–1.96)	<0.001	
Physical Activity				
High	3730	1.45 (1.21–1.74)	<0.001	0.795
Low	4388	1.71 (1.51–1.93)	<0.001	
Moderate	1109	1.60 (1.09–2.36)	0.019	
Race/Ethnicity				
Mexican American	1459	1.65 (1.30–2.11)	<0.001	0.992
Non-Hispanic Black	1962	1.53 (1.23–1.90)	<0.001	
Non-Hispanic White	3389	1.62 (1.41–1.87)	<0.001	
Other Hispanic	925	1.80 (1.48–2.20)	<0.001	
Other Race	1492	1.30 (0.99–1.70)	0.056	
Smoking Status				
Current	1478	1.65 (1.35–2.00)	<0.001	0.157
Former	2028	1.67 (1.33–2.10)	<0.001	
Never	4609	1.43 (1.21–1.69)	<0.001	

## Discussion

In this nationally representative study of U.S. adults, we observed a significant and graded association between depressive symptom severity and severe vision difficulty. Participants with higher PHQ-9 scores had progressively greater odds of reporting severe vision difficulty, even after adjustment for a broad set of sociodemographic factors, lifestyle behaviors, and chronic conditions. In the fully adjusted model, compared with participants with no depressive symptoms, those with mild, moderate, moderately severe, and severe depressive symptoms had progressively higher odds of severe vision difficulty. When the PHQ-9 score was analyzed as a continuous variable, each five-point increase was associated with 60% higher odds of severe vision difficulty. In addition, restricted cubic spline analysis showed a statistically significant overall association, whereas the test for non-linearity was not significant, suggesting that the association was largely close to linear across the observed PHQ-9 range.

Our findings are broadly consistent with previous epidemiological studies reporting a close relationship between visual impairment and adverse mental health outcomes, while extending the literature in several ways. First, using NHANES 2013-2018, we examined this association in a large nationally representative sample of U.S. adults. Second, by classifying PHQ-9 scores into five severity groups, we demonstrated a graded pattern across increasing levels of depressive symptom severity rather than relying on a simple dichotomous classification. Third, the combined use of categorical, continuous, and spline-based analyses allowed us to characterize the association more comprehensively. Notably, the spline analysis suggested that the relationship was primarily near-linear rather than strongly non-linear across the observed score range.

Several biologically and behaviorally plausible mechanisms may underlie the observed association, although these pathways were not directly examined in the present study. Prior literature has suggested that depression-related processes may be linked to systemic inflammation, endothelial dysfunction, oxidative stress, and microvascular abnormalities, all of which have also been implicated in ocular disease [16-19]. Individuals with depressive symptoms often exhibit elevated levels of pro-inflammatory cytokines such as interleukin-6 and tumor necrosis factor-α [20-22], and inflammatory pathways have been implicated in the development of vision-threatening ocular conditions, including age-related macular degeneration, diabetic retinopathy, and glaucoma [23,24]. In addition, depression-related processes have been associated with endothelial dysfunction, oxidative stress, and impaired microvascular circulation, which may further affect retinal perfusion and visual function [25-27]. Behavioral pathways may likewise be relevant, as greater depressive symptom burden may be associated with lower physical activity, poorer treatment adherence, and reduced health-seeking behavior. At the same time, psychosocial processes may also contribute, including the possibility that individuals with more severe depressive symptoms perceive or report functional visual limitations more negatively. Accordingly, these mechanisms should be regarded as tentative hypotheses rather than explanations directly supported by the current data.

This study has several strengths. The use of NHANES data provided a large and nationally representative sample of U.S. adults. All analyses accounted for the complex survey design, allowing population-level estimation. In addition, the use of both categorical and continuous PHQ-9 measures, together with restricted cubic spline modeling, enabled a more detailed characterization of the association between depressive symptom severity and severe vision difficulty. The subgroup analyses also suggested that the direction of association was generally similar across the examined strata; however, these analyses should be interpreted as exploratory.

Several limitations should be acknowledged. First, the cross-sectional design precludes causal inference, and the temporal ordering of depressive symptoms and severe vision difficulty cannot be determined. The observed association may be bidirectional: severe vision difficulty may contribute to psychological distress through reduced independence and social participation, whereas greater depressive symptom burden may amplify the perception or reporting of functional visual limitations. Second, severe vision difficulty was assessed using a self-reported questionnaire item asking whether the participant was blind or had serious difficulty seeing even when wearing glasses. Although this measure captures real-world functional vision, it combines heterogeneous visual states and may not map directly onto clinical ophthalmic diagnoses. However, self-reported functional vision has been widely used in population-based research [28]. Third, despite adjustment for multiple covariates, residual confounding remains possible, particularly from factors not fully captured in the available data, such as specific ocular diseases, psychotropic medication use, anxiety-related symptoms, sleep problems, and other behavioral or clinical characteristics. Finally, substantial exclusions due to missing covariate data may have introduced selection bias and reduced precision. Therefore, the findings should be interpreted cautiously, and future longitudinal studies using more clinically specific ophthalmic measures are needed to clarify temporality and underlying mechanisms.

## Conclusions

In this nationally representative cross-sectional study of U.S. adults, greater depressive symptom severity was significantly associated with higher odds of severe vision difficulty. This association showed a graded pattern across PHQ-9 categories and was also observed when the PHQ-9 score was analyzed as a continuous variable. Restricted cubic spline analysis further suggested that the association was largely close to linear across the observed PHQ-9 range. Given the cross-sectional design, these findings should be interpreted as evidence of association rather than causation. Future longitudinal studies incorporating objective ophthalmologic measures are needed to clarify temporality and the mechanisms underlying this relationship.
